# Development of a platform process for the production and purification of single‐domain antibodies

**DOI:** 10.1002/bit.27724

**Published:** 2021-03-25

**Authors:** Laura E. Crowell, Chaz Goodwine, Carla S. Holt, Lucia Rocha, Celina Vega, Sergio A. Rodriguez, Neil C. Dalvie, Mary K. Tracey, Mariana Puntel, Andrés Wigdorovitz, Viviana Parreño, Kerry R. Love, Steven M. Cramer, J. Christopher Love

**Affiliations:** ^1^ Koch Institute for Integrative Cancer Research Massachusetts Institute of Technology Cambridge Massachusetts USA; ^2^ Department of Chemical Engineering Massachusetts Institute of Technology Cambridge Massachusetts USA; ^3^ Department of Chemical and Biological Engineering Rensselaer Polytechnic Institute Troy New York USA; ^4^ Center for Biotechnology and Interdisciplinary Studies Rensselaer Polytechnic Institute Troy New York USA; ^5^ Instituto de Virología, Investigación en Ciencias Veterinarias y Agronómicas Instituto Nacional de Tecnología Agropecuaria Buenos Aires Argentina; ^6^ Department of Biological Engineering Massachusetts Institute of Technology Cambridge Massachusetts USA

**Keywords:** integrated purification, *Komagataella phaffii*, *Pichia pastoris*, single‐domain antibodies, straight‐through chromatography, VHH

## Abstract

Single‐domain antibodies (sdAbs) offer the affinity and therapeutic value of conventional antibodies, with increased stability and solubility. Unlike conventional antibodies, however, sdAbs do not benefit from a platform manufacturing process. While successful production of a variety of sdAbs has been shown in numerous hosts, purification methods are often molecule specific or require affinity tags, which generally cannot be used in clinical manufacturing due to regulatory concerns. Here, we have developed a broadly applicable production and purification process for sdAbs in *Komagataella phaffii* (*Pichia pastoris)* and demonstrated the production of eight different sdAbs at a quality appropriate for nonclinical studies. We developed a two‐step, integrated purification process without the use of affinity resins and showed that modification of a single process parameter, pH of the bridging buffer, was required for the successful purification of a variety of sdAbs. Further, we determined that this parameter can be predicted based only on the biophysical characteristics of the target molecule. Using these methods, we produced nonclinical quality sdAbs as few as 5 weeks after identifying the product sequence. Nonclinical studies of three different sdAbs showed that molecules produced using our platform process conferred protection against viral shedding of rotavirus or H1N1 influenza and were equivalent to similar molecules produced in *Escherichia coli* and purified using affinity tags.

## Graphical abstract

Unlike conventional antibodies, single‐domain antibodies (sdAbs) do not benefit from a platform manufacturing process, meaning that each new sdAb requires extensive process development to be produced and purified. We present here a platform process which can be used to produce phase‐appropriate, tag‐less sdAbs with nonclinical quality as few as five weeks after obtaining the target product sequence, and show that molecules produced using this process are comparable to the same sdAbs produced using affinity tag chromatography in nonclinical studies.

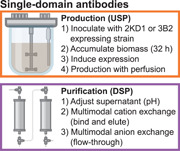

## 1 INTRODUCTION

Recombinant proteins, including enzymes, cytokines, hormones, antibodies, and antibody derivatives, are used to treat cancer, autoimmune disorders and rare diseases throughout the world. Development timelines for such biologic drugs are shrinking, particularly with regard to manufacturing process development (Baaj et al., 2017). Shorter development timelines may enable new drugs to reach patients sooner and allow biopharmaceutical companies to secure first‐to‐market status.

For some classes of molecules, such as monoclonal antibodies, platform manufacturing processes have emerged to reduce the time and effort required to develop a manufacturing process for a new molecule. In a platform process, the overall unit operations and order of these operations are standardized. Each new product, therefore, requires only minimal optimization of these steps. Biopharmaceutical pipelines are becoming more diverse, however, and other classes of biologics generally require unique production and purification processes, even for similar molecules (Morrison, 2020). Developing processes for these nonplatform biologics requires significant time and effort, limiting the number of molecules that can be manufactured for nonclinical or clinical use.

We have previously shown that a holistic approach to process development, coupled with a bench‐scale, integrated manufacturing platform, can reduce the time required to produce nonclinical material for a new biologic product to as few as 12 weeks after obtaining a target product sequence (Crowell et al., 2018). Further improvements to the process development timeline could be possible through the development of predictive or platform approaches to the manufacture of different classes of molecules, particularly in the chromatographic purification.

Single‐domain antibodies (sdAbs) are a class of molecules for which a platform approach could be developed. sdAbs offer the affinity and therapeutic value of conventional antibodies with increased stability and solubility (Arbabi‐Ghahroudi, 2017). Their small size (12–15 kDa) allows them to bind to previously intractable targets and their increased stability presents the potential for oral dosage (for intestinal diseases) (Harmsen & Haard, 2007). Significant scientific research has been conducted on sdAbs since their discovery in the early 1990s (Hamers‐Casterman et al., 1993), and recent events have highlighted the therapeutic potential of this class of molecules. Cablivi, an sdAb for the treatment of acquired thrombotic thrombocytopenic purpura, was approved by the FDA in 2019, and numerous others are in development (Morrison, 2019). Single‐domain antibody therapies are also currently being investigated in the treatment of the novel coronavirus SARS‐CoV‐2 (Huo et al., 2020; Wrapp et al., 2020).

As a class of molecules, sdAbs are similar to monoclonal antibodies in that they have a generally conserved structure and similar biophysical characteristics. Unlike conventional antibodies, however, sdAbs do not benefit from a platform manufacturing process. Although sdAbs have successfully been produced in a variety of different hosts including bacteria, yeast, mammalian, plant and insect cells (Liu & Huang, 2018), platform purification methods for such sdAbs are not widespread. Affinity tags often used for the purification of sdAbs, such as His tags, can impact protein folding, stability, solubility, and aggregation and therefore present risks of immunogenicity (Khan et al., 2012; Wu & Filutowicz, 1999). Due to the increased risk of immunogenicity, the use of such affinity tags in the purification of therapeutic proteins is strongly discouraged by regulatory agencies. While some sdAbs will interact with protein A resins, an affinity resin commonly used in the purification of monoclonal antibodies, this interaction is not universal (De Genst et al., 2006). Efforts have been made to engineer the protein sequences of sdAbs that do not bind to protein A to improve binding, but this requires sequence modifications, which could affect the affinity and immunogenicity of the target molecule (Henry et al., 2016). It would likely be possible to create an affinity resin specific to sdAbs using methods similar to those used to develop an affinity resin for recombinant factor FVIII (McCue et al., 2009). The cost and reliability of supply for custom affinity resins can be prohibitive, however. A predictable process for the purification of sdAbs based on nonaffinity methods could simplify the manufacturing of these products and enable rapid translation from sequence to nonclinical material.

A nonaffinity purification process has been developed for sdAbs utilizing a new chromatographic method known as void‐exclusion anion exchange (VEAX), followed by multimodal cation exchange (Fan et al., 2019). The optimal loading volume for the VEAX step reported was <0.18 column volumes, however, indicating that very large columns and buffer volumes would be required for this process. A platform purification process for sdAbs utilizing more traditional unit operations would be more space and resource efficient.

Here, we demonstrate the rapid development of integrated production and purification processes for eight sdAbs. Further, we present a method for the initial production of a new sdAb based only on its biophysical characteristics. We use this methodology to produce initial nonclinical batches of products in as few as 5 weeks after obtaining the product sequence. Finally, we show that molecules produced using our platform process are equivalent to similar molecules produced in *Escherichia coli* and purified using a His tag in nonclinical studies.

## 2 MATERIALS AND METHODS

### 2.1 Single‐domain antibody sequences

Product sequences were received from the Parreño lab at the Instituto Nacional de Tecnología Agropecuaria. All products were nonglycosylated, llama‐derived sdAbs (VHH). Products included two neutralizing group A rotavirus (RVA) sdAbs, 2KD1 and 3B2 (Garaicoechea et al., 2008), five norovirus‐specific sdAbs, N1–N5 (Garaicoechea et al., 2015), and two H1N1 influenza‐specific sdAbs, G41 and E13 (Parreño lab, unpublished). For the norovirus sdAbs, N1, N2, and N5 are GII.4‐specific (denoted as M1, M4, and M6 in Garaicoechea et al., 2015, respectively), and N3 and N4 are GI.1‐specific (denoted as N1 and N2 in Garaicoechea et al., 2015, respectively). Both H1N1 influenza‐specific sdAbs were obtained from an immune library derived from a llama immunized with the H1N1 influenza vaccines used in humans. Excess histidines (traditionally used for His tag) were removed from all sequences.

### 2.2 Strain generation and protein production

Wild‐type *Komagataella phaffi* (NRRL Y‐11430) was modified to express the sdAb of interest as described previously (Crowell et al., 2018). InSCyT bioreactors were used for protein production as described previously (Crowell et al., 2018) using rich defined media (Matthews et al., 2017). A total of 4% glycerol was added for outgrowth and 5% methanol was added for production. A total of 30 g/L sorbitol was also added during production. In the bioreactor, temperature, pH, and dissolved oxygen were maintained at 25°C, 6.5, and 25%, respectively. Additional supernatant for characterization of the purification process was produced using shake cultivations as described previously (Timmick et al., 2018), except rich defined media was used (Matthews et al., 2017). All chemical reagents were purchased from Sigma‐Aldrich.

### 2.3 Protein purification

Protein purification was carried out on the purification module of the InSCyT system as described previously (Crowell et al., 2018). The resins used included the multimodal cation exchanger CMM HyperCel and the salt tolerant anion exchanger HyperCel STAR AX. All columns were equilibrated in the appropriate buffer before each run. Product‐containing supernatant was adjusted to pH 5.0 using 15 mM citric acid. The adjusted supernatant was loaded onto a prepacked CMM HyperCel column (1‐ or 5‐ml) (Pall Corporation), re‐equilibrated with 20 mM sodium citrate pH 5.0, washed with 20 mM sodium phosphate pH 6.0, and eluted with 20 mM sodium phosphate pH 7.0 or 8.0, 100 mM NaCl. Eluate from column 1 above 15 mAU was flowed through a 1‐ml prepacked HyperCel STAR AX column (Pall Corporation). Flow‐through from column 2 above 15 mAU was collected.

pH gradient screens on CMM HyperCel were carried out using an AKTA Explorer 10 system (GE Healthcare Life Sciences) equipped with a Frac‐950 fraction collector and a P‐960 sample pump and controlled using Unicorn 5.1 software. Step changes in pH were examined including pH 7.0, 8.0, 9.0, and 10.0, all at 100 mM NaCl. Based upon the elution pH identified, a salt gradient was then run at that pH from 0 to 1 M NaCl.

### 2.4 Analytical procedures

Wet cell weight was determined as described previously (Crowell et al., 2018). Sample concentrations were determined by measuring the absorbance at 280 nm. Sodium dodecyl sulfate polyacrylamide gel electrophoresis (SDS‐PAGE) was carried out under reducing conditions using Novex 12% Tris‐glycine Gels or Novex 16% Tricine Gels (Thermo Fisher Scientific) according to the manufacturer's recommended protocol and stained using Instant Blue Protein Stain (Thermo Fisher Scientific). Samples were analyzed for host cell‐protein content using the *Pichia pastoris* 1st generation HCP ELISA kit from Cygnus Technologies according to the manufacturer's recommended protocol. Samples were analyzed for residual host‐cell DNA using the Quant‐iT dsDNA High‐Sensitivity Assay Kit (Invitrogen) according to the manufacturer's protocol except the standard curve was reduced to 0–20 ng. Unpurified samples were not analyzed for DNA content due to interference of media components with the Quant‐iT dsDNA High‐Sensitivity Assay Kit. Instead, typical DNA content of unpurified material produced in *Komagataella phaffii* is used for comparison (Timmick et al., 2018). Purification yields were calculated using concentration measurements at 280 nm (purified samples) or estimated from SDS‐PAGE (unpurified samples).

### 2.5 Nonclinical studies

#### 2.5.1 Rotavirus

SdAbs 2KD1 and 3B2 produced on the InSCyT system were compared with the same molecules expressed with a His tag in *E. coli* in 10 L bioreactors and purified using immobilized metal affinity chromatography (IMAC). The binding affinity of each clone to rotavirus A (RVA) particles was studied using ELISA. Briefly, 96‐well plates (NUNC‐Maxisorp) were coated at 37°C with a RVA specific bovine polyclonal IgG serum (1:5000) and blocked with 10% nonfat milk (prepared in PBS‐Tween 20 0.5%). Bovine RVA (UK strain G6P[5]; 10^7^ FFU/ml) or mock‐infected MA‐104 cell supernatant fluids were added for another hour at 37°C. Serial 10‐ or 4‐fold dilutions were assayed for both clones starting at an initial protein concentration of 0.1 mg/ml. This step was followed by incubation with a 1:3000 dilution of a rabbit hyperimmune serum against sdAbs and then with a commercial horseradish peroxidase (HPR) labeled goat polycolonal Ab to rabbit IgG (1:2000; KPL) for 1 h at 37°C. Commercial hydrogen peroxide and ABTS (Sigma‐Aldrich) were used as the substrate/chromogen system and the reaction was stopped with 5% SDS. Optical density was measured at 405 nm. Dose–response curves were modeled by fitting a four‐parameter sigmoidal dose–response curve using GraphPad Prism 7 and results were expressed as the effective dose 50% (EC50).

The protective effects of the InSCyT produced 2KD1 and 3B2 were compared to the *E. coli* produced products in a suckling mouse model of murine RVA infection and disease. Three‐day old sucking mice were randomly distributed into six groups of six mice each. Treatment was administered as 100 µg in 100 µl once per day for 5 days (Days −1, 0 (challenge day), 1, 2, 3). Groups 1–4 received InSCyT 2KD1, InSCyT 3B2, *E. coli* 2KD1, and *E. coli* 3B2, respectively. Groups 5 and 6 received 0.9% NaCl (#PR 107/19). All materials were sterile filtered before treatment. All groups except for group 5 were orally challenged with 450 FFU of murine rotavirus (Ecw # 953/18) on Day 0. Viral inoculation and all treatments were administered using a flexible intragastric gauge. All mice were examined daily to assess the occurrence of diarrhea by gentle abdominal palpation and collection of feces. Mice were euthanized on Day 8 and the intestines were disaggregated in minimal essential medium (Invitrogen). Rotavirus shedding was evaluated in each macerate by ROTADIAL sdAb capture ELISA and viral infectivity titer was determined using a cell culture immunofluorescence assay and expressed in fluorescent focus forming units (FFFU/ml). An sdAb labeled with Alexa Fluor 488 was used to detect RVA‐infected cells and fluorescent cells were counted using a fluorescence microscope.

#### 2.5.2 Influenza

E13 produced on the InSCyT system was compared with the same molecule expressed with a His tag in *E. coli* in and purified using IMAC. Six week old female BALB/c mice were randomly distributed into three groups of 10 animals. Groups were treated with 5 mg/kg in 100 µl of InSCyT E13, *E. coli* E13, or 0.9% NaCl via intraperitoneal administration. All materials were sterile filtered before treatment. Four hours after treatment, all animals were intranasally infected with 50 µl of H1N1ma virus (2DL50). Body weight and survival were monitored daily for 13 days postinfection. Animals showing a loss of weight greater than or equal to 30% of their total body mass and general inactivity were euthanized. Five animals from each group were euthanized 4 days postinfection to assess the viral titer in the lungs. Lungs were homogenized by mechanical methods using sand and glass rod. The viral titer was determined by infection of Madin Darby canine kidney cells. The median tissue culture infections dose (TCID50) and median lethal dose (LD50) of H1M1ma virus were determined by Reed and Muench Method.

## 3 RESULTS AND DISCUSSION

We set out to demonstrate the production of two rotavirus‐specific sdAbs, 3B2 and 2KD1 (Garaicoechea et al., 2008), on our bench‐scale, integrated manufacturing platform (InSCyT) (Crowell et al., 2018). These sdAbs bind to the VP6 protein of RVA and have previously been shown to confer partial protection against severe diarrhea and significantly reduce virus shedding in mice (Maffey et al., 2016). Both products expressed well in small‐scale batch cultivations from *K. phaffii*. Noting that the biophysical characteristics of these sdAbs were similar to other products we have made on the InSCyT system (Crowell et al., 2018), we predicted fermentation conditions based on previous experience. Based on the in silico purification prediction tool by Timmick et al. (2018), we determined that both sdAbs could be purified using exactly the same two‐column, integrated, straight‐through process. Straight‐through chromatography, where the eluate of one column is loaded directly onto the next column without any changes to the pH or conductivity of the buffer, is an integrated manufacturing technique which removes the need for hold tanks and additional unit operations, significantly reducing manufacturing footprint, buffer usage, and processing time (Andersson et al., 2017; Löfgren et al., 2019). The straight‐through process predicted for the purification of the rotavirus sdAbs comprised a bind‐and‐elute capture step on CMM HyperCel resin, a multimodal cation exchanger, and a flowthrough polishing step on HyperCel STAR AX resin, a salt‐tolerant anion exchanger.

**Figure 1:**
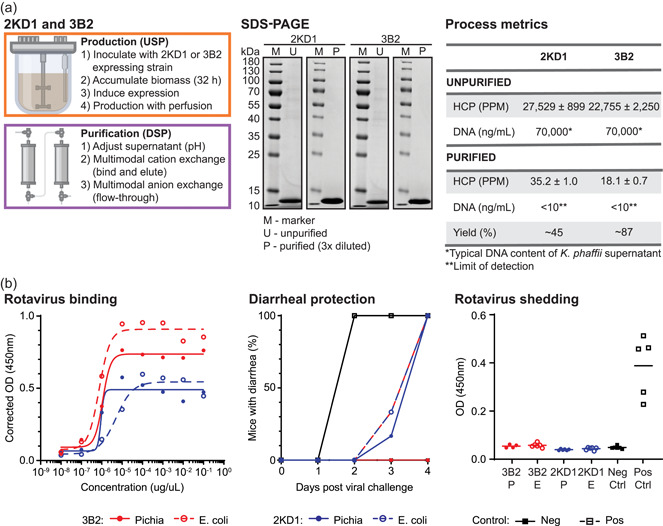
Production and purification of rotavirus specific single‐domain antibodies, 2KD1 and 3B2, on the InSCyT system. (a) Process flowchart (left) and product‐quality analyses (center and right) for the production of 2KD1 and 3B2. SDS‐PAGE (12% Tris‐glycine) analysis of unpurified (U) and purified (P) samples. Quantification of yield, HCP, and DNA in unpurified and purified samples. DNA content of unpurified samples is represented as the typical DNA content of *Komagataella phaffii* supernatant as determined from Timmick et al. (2018) (see Section 2). Error represents the range of technical triplicates. (b) Analysis of in vitro binding and in vivo protection of InSCyT‐produced 2KD1 and 3B2 as compared to the same molecules produced in *Escherichia coli* and purified using a His tag. Dose response curve for 2KD1 and 3B2 binding to RVA (left), diarrheal protection (center), and rotavirus shedding (right) in mice after oral challenge with 450 FFU of murine rotavirus. HCP, host‐cell protein; M, molecular mass marker; PPM, parts per million; SDS‐PAGE, sodium dodecyl sulfate polyacrylamide gel electrophoresis [Color figure can be viewed at wileyonlinelibrary.com]

We deployed the production process for both 2KD1 and 3B2 using the InSCyT system. We assessed the purified sdAbs for quality attributes required for nonclinical use, including identity, safety, and purity. SDS‐PAGE was used to confirm identity, and showed that the purified products were both of the expected molecular weight, 13.5 kDa for 2KD1 and 13.8 kDa for 3B2 (Figure 1a). Key contributors to product safety and purity include the presence of process‐related impurities, including host‐cell proteins (HCPs) and host‐cell DNA, and the levels of potentially immunogenic product‐related impurities, such as aggregates. Levels of process‐related impurities were each below typical values for clinical‐stage development (1000 PPM for HCPs and 10 ng/dose for DNA) (Jawa et al., 2016; The European Agency for the Evaluation of Medicinal Products, 1997; World Health Organization, 2013). Regulatory agencies usually consider limits for HCPs on a case‐by‐case basis, although in vitro studies using peripheral blood mononuclear cells from both healthy and diseased individuals have shown that HCP levels up to 4000 PPM from Chinese hamster ovary (CHO) cells do not pose a higher immunogenicity risk than a highly purified monoclonal antibody (<50 PPM) (Jawa et al., 2016). HCPs were reduced below 100 PPM and DNA was below the limit of detection of our assay (10 ng/ml) for both molecules (Figure 1a). Purification yields were approximately 45% and 87% for 2KD1 and 3B2, respectively (Figure 1a). We did observe dual bands on the SDS‐PAGE suggesting the presence of product‐related impurities; we further characterized these bands via MALDI as *N*‐terminal truncation (data not shown). Given the lack of evidence on the immunogenicity of such *N*‐terminal truncations, we determined the clearance of process‐related impurities by our purification process made this material phase‐appropriate for nonclinical studies. Further engineering of the expression vector with alternative signal sequences could alleviate the expression of this variant if needed (Gibson et al., 2017).

Nonclinical studies were carried out with each purified 2KD1 and 3B2 and compared to similar material produced in *E. coli* and purified using a *C*‐terminal His tag. Both molecules produced using our process recognized rotavirus particles in an ELISA (Figure 1b). The 50% effective dose (EC50) for our 3B2 (1.11e^−4^ µg/µl) was not significantly different than the 3B2 produced in *E. coli* (7.43e^−5^ µg/µl) (*p* = .461). The EC50 for our 2KD1 (8.99e^−5^ µg/µl) was significantly lower than the *E. coli* produced 2KD1 (5.01e^−4^ µg/µl), indicating that our 2KD1 binds to rotavirus particles at lower concentrations (*p* = .002). This may be due to the *N*‐terminal truncations observed in our product, or to the presence of the *C*‐terminal His tag on the *E. coli*‐produced product, which has been shown to impact the structure and function of other protein antigens (Khan et al., 2012); further testing is required to confirm either hypothesis. After confirming the binding affinities of the sdAbs produced using our process, we tested their ability to induce protection against diarrhea and rotavirus shedding in mice (Figure 1b). There was a delay in the onset of diarrhea for all treated mice, and mice treated with our 3B2 did not develop diarrhea after 4 days. Further, both molecules produced using our process induced protection against virus shedding as measured 4 days postinoculation, which was comparable to the products produced in *E. coli*. Overall, both molecules produced on the InSCyT system using our initial processes were comparable to those produced from *E. coli*‐based expression and IMAC purification.

**Figure 2:**
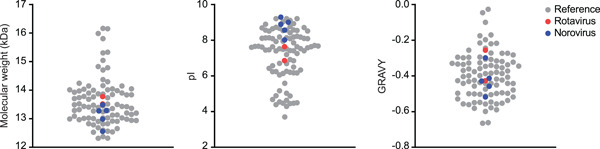
Comparison of the biophysical characteristics of the sdAbs examined in this study (rotavirus specific sdAbs, red; norovirus specific sdAbs, blue) along with a reference data set of sdAbs (gray) obtained from Mitchell and Colwell (2018). GRAVY, grand average of hydropathy; pI, isoelectric point [Color figure can be viewed at wileyonlinelibrary.com]

Based on our success using a single process to produce both 2KD1 and 3B2, we hypothesized that the selected resins could be used to purify a wide range of sdAbs. To test this hypothesis, we obtained the sequences for five additional sdAbs specific to Norovirus (Garaicoechea et al., 2015). We compared the biophysical properties of these five sdAbs to our original two products, along with other known sdAb sequences (Mitchell & Colwell, 2018). We confirmed that our set of sdAbs was representative of a wide range of molecules in this class based on fundamental biophysical features, including molecular weight, isoelectric point (pI) and hydrophobicity, measured as GRAVY score (Figure 2).

Based on the observed range of pI, we predicted our purification process would require minor adjustments to the operating conditions to apply more broadly. Specifically, we expected that the conditions of the bridging buffer—the buffer used to transition from one column to another column in straight‐through chromatographic processes—would need to be adjusted to guarantee elution from the first column. In this case, the bridging buffer is the buffer used to elute the product from the capture column and to flow the product through the polish column. We performed pH and salt gradient screens on each of the sdAbs to determine the conditions required to elute the product from the CMM resin (Table 1). Based on these results, we developed two processes, differing only in the pH of the bridging buffer, predicted to remove process‐related impurities from six of the seven sdAb products.

**Table 1:** Biophysical characteristics and bridging buffer conditions required to elute each sdAb from the capture column, CMM HyperCel

Molecule	Target	pI	GRAVY	Variable region pI	Bridging pH	Bridging salt (mM)
2KD1	Rotavirus	6.87	−0.425	3.74	7.0	100
3B2	Rotavirus	7.65	−0.255	4.04	7.0	100
N1	Norovirus	8.03	−0.298	5.14	7.0	100
N2	Norovirus	9.30	−0.517	10.34	8.0	800
N3	Norovirus	8.91	−0.412	8.77	8.0	100
N4	Norovirus	9.02	−0.428	6.38	7.0	100
N5	Norovirus	8.58	−0.457	8.65	8.0	100

For the remaining product (N2), the experimental gradient screens showed that a very high salt concentration (800 mM) was required for elution from the CMM resin. This high salt concentration precludes the use of a straight‐through purification process as DNA removal is not achieved on the polishing column at these conditions. This molecule is therefore outside of the applicable range for our platform process, and was not included in additional experiments. Notably, this molecule had the highest pI of any of the molecules we examined, indicating that it may be possible to set limits on the applicability of our platform process based on biophysical features. Given the high pI, it is unlikely this molecule would be selected for development based on typical developability assessments (Xu et al., 2019).

**Figure 3:**
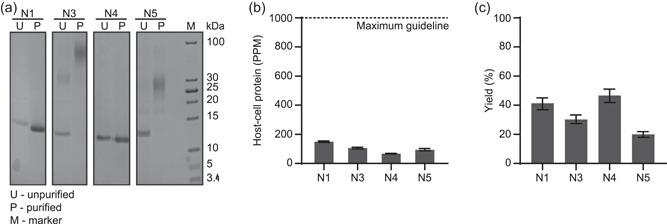
Production and purification of norovirus specific single‐domain antibodies on the InSCyT system. (a) SDS‐PAGE (16% tricine) analysis of unpurified (U) and purified (P) samples. (b) Quantification of HCP in purified samples compared to the maximum guideline for clinical‐stage development (Jawa et al., 2016; The European Agency for the Evaluation of Medicinal Products, 1997). Error bars represent the range of technical triplicates. (c) Purification yields for each process. Error bars represent a relative *SD* of 10%. HCP, host‐cell protein; M, molecular mass marker; PPM, parts per million; SDS‐PAGE, sodium dodecyl sulfate polyacrylamide gel electrophoresis

We then tested our predicted production and purification processes on the other four norovirus sdAbs (Figure 3). The pH of the bridging buffer was 7.0 for N1 and N4 and 8.0 for N3 and N5. In all cases, HCPs were reduced to <200 PPM (HCP for unpurified samples ranged from 176,000 ± 6000 to 612,000 ± 25,000 PPM) and DNA was reduced below the limit of detection of our assay. Approximate yields ranged from 20% to 46%. As will be described below, we have also developed methods for optimizing the purification of each individual molecule to significantly improve the yields (Crowell et al., submitted). Aggregation and dimerization were observed in the purified samples of N3 and N5, respectively (Figure 3a). Nevertheless, we were successfully able to recover the product and remove process‐related variants for all four of these additional sdAbs using the same platform process with a minor adjustment to the bridging buffer.

Product‐related impurities such as aggregates can be immunogenic and adversely affect the safety of a product. Size‐exclusion analysis of cell culture fluid containing unpurified N3 and N5 showed that the aggregation began before purification, and was further exacerbated during purification, indicating that these specific sdAb sequences (N3 and N5) may be particularly prone to aggregation (data not shown). These data suggest that our platform process for production and purification of sdAbs could be used to rapidly determine whether specific products are prone to aggregation. As demonstrated here, multiple sequences can easily be produced and purified, and sequences with minimal product‐related impurities can be selected for further nonclinical testing.

To eliminate the need for a gradient screen on the capture resin for future products, we attempted to use the biophysical properties of the target proteins to predict the best pH for the bridging buffer. We determined the Pearson correlation coefficient between the pI or GRAVY score and the bridging buffer pH for the molecules we had already successfully purified. Given the conserved structure of sdAbs as a class of molecules (Sircar et al., 2011), we also examined the pI and GRAVY score for relevant subsets of the overall sequence, including the variable (or CDR) and framework regions (Table 2).

**Table 2:** Pearson correlation coefficient (*r*) for sequence specific biophysical traits (pI or GRAVY) as compared to the pH of the bridging buffer (7.0 or 8.0)

	pI		GRAVY	
Sequence region	Pearson's *r*	*p* value	Pearson's *r*	*p* value
Full sequence	.671	.099	−.714	.071
Framework	.574	.178	−.859	.013
Variable region (H1, H2, and H3)	.923	.003*	−.309	.501
Variable region (H1 or CDR1)	.582	.170	−.168	.719
Variable region (H2 or CDR2)	.662	.105	.418	.351
Variable region (H3 or CDR3)	.962	.001*	−.459	.300

*Note*: Sequence regions were determined as described in Sircar et al. (2011).

* *p* < .005.

**Figure 4:**
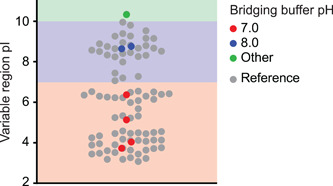
Comparison of the pI of the variable region for sdAbs examined in this study along with a reference data set of sdAbs (gray) obtained from Mitchell and Colwell (2018). sdAbs from this study are colored by the pH of the bridging buffer in their purification process (7.0, red; 8.0, blue). N2, the sdAb for which our purification process was not applicable is colored green. Background colors correspond to the guidelines proposed in Equation (1) for predicting the pH of the bridging buffer (7.0, red; 8.0, blue; and process not applicable, green). pI, isoelectric point [Color figure can be viewed at wileyonlinelibrary.com]

After correcting for multiple hypothesis testing using a simple Bonferroni correction (Dunn, 1961), we determined that the pI of the variable region (all parts) and the pI of the H3 (or CDR3) region, in particular, were significantly correlated with bridging buffer pH (Table 2). Interestingly, the pI of the overall molecule and the framework pI were not significantly correlated with bridging buffer pH, indicating that the variable regions are playing the most important role in resin binding. This result is not particularly surprising as this is also the region that is most critical for antigen binding (Mitchell & Colwell, 2018). Comparing the pI of the variable region (Var_pI_) of each of the molecules we had successfully purified thus far, we developed criteria for determining the bridging buffer pH for a new sdAb molecule based on sequence alone (Figure 4 and Equation 1). Notably, the pI of the variable region of N2, the molecule for which our platform process was not applicable, was very high (10.34). We therefore believe that the pI of the variable region can be used to determine both whether our process will be applicable for a given sdAb, and what the pH of the bridging buffer should be.

(1) BridgingBufferpH(VarpI)=7.0,VarpI<7.08.0,7.0<VarpI<10.0N/AVarpI>10.0.

**Figure 5:**
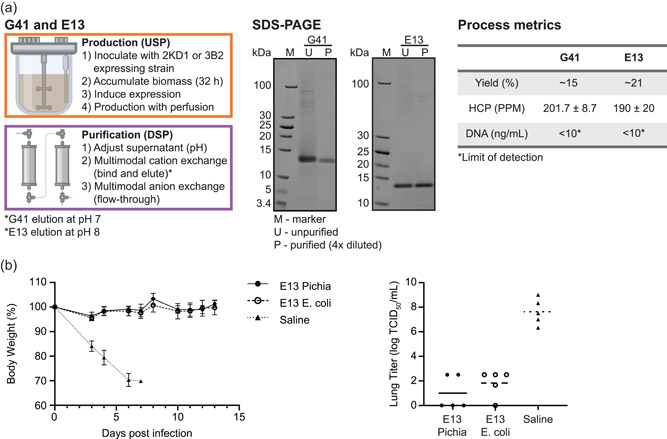
Production and purification of influenza specific single‐domain antibodies, G41 and E13, on the InSCyT system. (a) Process flowchart (left) and product‐quality analyses (center and right) for the production of G41 and E13. SDS‐PAGE (16% tricine) analysis of unpurified (U) and purified (P) samples. Quantification of yield, HCP, and DNA in purified samples. Error represents the range of technical triplicates. (b) Analysis of in vivo protection of InSCyT‐produced E13 as compared to the same molecules produced in *Escherichia coli* and purified using a His tag. Change in body weight after infection (left) and viral titer in lung homogenates analyzed 4 days after viral infection. Error bars represent the range across five animals. HCP, host‐cell protein; M, molecular mass marker; SDS‐PAGE, sodium dodecyl sulfate polyacrylamide gel electrophoresis [Color figure can be viewed at wileyonlinelibrary.com]

To test our proposed guideline, we next obtained the sequences for two additional nanobodies, this time specific to H1N1 influenza. We predicted purification processes for these two sdAbs based only on the target sequence. We predicted an elution pH of 7.0 would be successful for G41 (Var_pI_ = 3.96) and an elution pH of 8.0 would be successful for E13 (Var_pI_ = 8.89). We deployed full production and purification processes for these molecules on the InSCyT platform within 5 weeks of obtaining the sequences. Both products were successfully recovered, HCPs were reduced below 250 PPM and DNA was reduced below the limit of detection for our assay (Figure 5a). Approximate purification yields, however, for the G41 and E13 processes were 15% and 21%, respectively.

To confirm that product quality was maintained using our platform production and purification process, we proceeded with nonclinical testing for E13. Similar to the rotavirus sdAbs, our E13 was compared to the same product produced in *E. coli* and purified using a His tag. We evaluated the prophylactic efficacy of E13 in the H1N1 murinized (ma) mouse model. Our E13 prevented weight loss and conferred protection against virus shedding in the lungs similar to the E13 produced in *E. coli* (Figure 5b).

While removal of process‐related variants was consistent in all cases discussed above, approximate yields for the purifications ranged from 15% to >85%. Although these yields may be acceptable for initial sdAb production for nonclinical testing to validate safety and efficacy, higher yields would likely be required for clinical and commercial production processes. We believe that the large variance in yield is due partly to varied dynamic binding capacities. Based on in‐process data, including UV traces, we observed breakthrough during capture column loading in multiple instances, indicating overloading. This occurred most often upon the initial InSCyT system runs for a new molecule, such as for G41 and E13, and particularly when titers in the bioreactor were significantly higher than expected. Furthermore, we believe that while initial process conditions can be predicted from sequence alone, molecule‐specific experimental optimization is likely still required to consistently achieve high yields. This is similar to the production of monoclonal antibodies, where platform processes are initially applied to produce a molecule for nonclinical testing, and then optimization is carried out to obtain a final, high‐yielding process for clinical and commercial manufacturing (Liu et al., 2010).

Since our process employs straight‐through purification, we could not use conventional methods to optimize the process, as these would optimize each column individually. We therefore deployed a method we developed for the optimization of buffer conditions in straight‐through purification processes, detailed in a companion paper (Crowell et al., submitted). Briefly, after resin selection, a two‐step optimization of buffer conditions was carried out. The first step included a series of range‐finding experiments on each individual column, similar to conventional screening. Potential operating regions for each resin were then overlaid to determine applicable regions for integrated operation. In the second step, a statistical model was developed for the fully integrated, multicolumn process using design of experiments based on the operating regions determined in the first step. This model was used to predict the buffer conditions to maximize yield while minimizing process‐related impurities.

Applying this optimization methodology to G41, the influenza sdAb whose purification process we had predicted only from its biophysical characteristics, we determined the optimal capture buffer to be pH 4.3 and 19 mS/cm and the optimal bridging buffer to be pH 6.8 and 10 mM NaCl. Using these optimized conditions, we were able to improve the yield from ~15% to 88%, with HCPs and DNA below relevant levels, in about 3 weeks (Crowell et al., submitted).

## 4 CONCLUSIONS

We have developed a platform process for the rapid production and purification of sdAbs and have demonstrated the production of eight different sdAb products at phase‐appropriate quality. Initial sdAb production using this platform process required determination of a single parameter, pH of the bridging buffer. We demonstrated here that this parameter can be predicted based only on the biophysical characteristics of the sdAb. Using these methods, we produced nonclinical quality sdAbs within 5 weeks of identifying the product sequence. Finally, we showed that molecules produced using our platform process are equivalent to similar molecules produced in *E. coli* and purified using a His tag in nonclinical studies.

The platform process for the production and purification of sdAbs described here allows a new product to be produced for nonclinical testing only 5 weeks after obtaining the target sequence. This rapid production enables the timely analysis of stability, safety, and efficacy with little effort required to develop a manufacturing process. Further, such rapid techniques may enable more molecules to be analyzed for these attributes because fewer resources are required to reach this stage for a new molecule. While only process‐related variants are explicitly removed in the proposed process, remaining product‐related variants could be analyzed for immunogenicity in nonclinical studies to determine whether removal is required. If removal of a specific product‐related variant is deemed necessary, a third column could be added to the chromatography process proposed here. Combined screening and in silico methods can also be used to design integrated purification processes for products with significant product‐related impurity removal challenges (Vecchiarello et al., 2019). Alternatively, changes could be made to the strain or the molecule itself to reduce the formation of this variant in the first place. After the safety, efficacy, and stability of a molecule are confirmed (or during the nonclinical testing to confirm these characteristics), the purification process can be further optimized to reach appropriate yields. We believe that the platform process described here could allow more sdAbs to reach the clinic, and ultimately patients.

## CONFLICT OF INTERESTS

Laura E. Crowell, Chaz Goodwine, Kerry R. Love, Steven M. Cramer, and  J. Christopher Love have filed patents related to the InSCyT system and methods. Andrés Wigdorovitz and Viviana Parreño have filed patents related to the product sequences used in this study. Kerry R. Love and J. Christopher Love are cofounders and consultants to Sunflower Therapeutics PBC.

## AUTHOR CONTRIBUTIONS

Laura E. Crowell, Chaz Goodwine, Kerry R. Love, Steven M. Cramer, and J. Christopher Love conceived and designed production and purification experiments. Neil C. Dalvie and Mary K. Tracey generated and characterized yeast strains. Sergio A. Rodriguez and Mary K. Tracey performed fermentations. Laura E. Crowell and Chaz Goodwine designed and performed protein purifications. Laura E. Crowell and Sergio A. Rodriguez performed quality assessments. Carla S. Holt, Lucia Rocha, Celina Vega, Mariana Puntel, and Viviana Parreño performed animal studies. Laura E. Crowell, Viviana Parreño, Kerry R. Love, Steven M. Cramer, andJ. Christopher Love wrote the manuscript. J. Christopher Love, Steven M. Cramer, Kerry R. Love, Viviana Parreño, Andrés Wigdorovitz, and Mariana Puntel designed the experimental strategy and supervised analysis. All authors reviewed the manuscript.

## Data Availability

The datasets generated and analyzed in this study are available from the corresponding author upon reasonable request.
